# Activity of Human Apurinic/Apyrimidinic Endonuclease APE1 Toward Damaged DNA and Native RNA With Non-canonical Structures

**DOI:** 10.3389/fcell.2020.590848

**Published:** 2020-10-30

**Authors:** Anastasia T. Davletgildeeva, Alexandra A. Kuznetsova, Olga S. Fedorova, Nikita A. Kuznetsov

**Affiliations:** ^1^Institute of Chemical Biology and Fundamental Medicine of the SB RAS, Novosibirsk, Russia; ^2^Department of Natural Sciences, Novosibirsk State University, Novosibirsk, Russia

**Keywords:** DNA repair, non-B-DNA, quadruplex, AP endonuclease, nucleotide recognition

## Abstract

The primary role of apurinic/apyrimidinic (AP) endonuclease APE1 in human cells is the cleavage of the sugar phosphate backbone 5′ to an AP site in DNA to produce a single-strand break with a 5′-deoxyribose phosphate and 3′-hydroxyl end groups. APE1 can also recognize and incise some damaged or modified nucleotides and possesses some minor activities: 3′–5′ exonuclease, 3′-phosphodiesterase, 3′-phosphatase, and RNase H. A molecular explanation for the discrimination of structurally different substrates by the single active site of the enzyme remains elusive. Here, we report a mechanism of target nucleotide recognition by APE1 as revealed by the results of an analysis of the APE1 process involving damaged DNA and native RNA substrates with non-canonical structures. The mechanism responsible for substrate specificity proved to be directly related to the ability of a target nucleotide to get into the active site of APE1 in response to an enzyme-induced DNA distortion.

## Introduction

Human apurinic/apyrimidinic (AP) endonuclease APE1 is the key enzyme of the base excision repair pathway, which is responsible for processing AP sites in DNA (Wilson and Barsky, 2001; Demple and Sung, 2005). In the alternative nucleotide incision repair pathway, APE1 makes an incision of the phosphodiester bond on the 5′ side of a damaged nucleotide, resulting in the formation of a 3′-OH group and a 5′ dangling damaged nucleotide (Ischenko and Saparbaev, 2002; Gros et al., 2004; Guliaev et al., 2004; Daviet et al., 2007; Christov et al., 2010; Vrouwe et al., 2011; Prorok et al., 2012, 2013). In addition, the APE1 enzyme possesses endoribonuclease (Barzilay et al., 1995; Berquist et al., 2008; Barnes et al., 2009), 3′-phosphodiesterase, 3′-phosphatase (Chen et al., 1991), and 3′–5′-exonuclease activities (Chou and Cheng, 2002; Kuznetsova et al., 2018a). Of note, a comparison of all these DNA lesions and natural DNA and RNA nucleotides makes it clear that they significantly differ from one another in chemical nature. On the one hand, such a variety of substrates indicates that the enzyme is indeed multifunctional; on the other hand, it raises the question of how APE1, which has a single active site, can control its substrate specificity and activity.

X-ray crystallographic studies of human APE1 in the free state (Gorman et al., 1997; Beernink et al., 2001; Manvilla et al., 2013) and in complex with nicked or abasic DNA (Mol et al., 2000a,b; Tsutakawa et al., 2013; Freudenthal et al., 2015) have revealed that this enzyme interacts preferentially with one of the duplex strands, usually to form hydrogen bonds and electrostatic contacts between DNA phosphate groups and amino acid side chains or amide groups of the peptide bonds of the protein. It has been shown that in a catalytically active complex with APE1, DNA is bent, and an abasic nucleotide is flipped out of the DNA double helix into the active site of the enzyme. As illustrated recently (Whitaker et al., 2018) in an elegant series of high-resolution APE1–DNA structural snapshots, APE1 removes 3′ nucleotides in the course of the 3′–5′-exonuclease reaction by placing the 3′ group within the intra-helical DNA cavity via a non–base-flipping mechanism. This process is facilitated by DNA structural disturbances caused by the presence of a mismatched or damaged base or nick formation as well as DNA bending. Nevertheless, the reality of this mechanism in endonuclease reactions is questionable due to significant restrictions on the nucleotide mobility in an unbroken DNA chain.

In our previous study (Kuznetsova et al., 2018b), we performed pulsed electron–electron double resonance (PELDOR) spectroscopy and pre–steady-state kinetic analysis with Forster resonance energy transfer (FRET) detection of DNA conformational changes during DNA binding and lesion recognition in model damaged duplex substrates containing 1,*N*^6^-ethenoadenosine, α-adenosine, 5,6-dihydrouridine, or an abasic site. Equilibrium PELDOR and kinetic FRET data indicated that DNA binding by APE1 leads to noticeable damage-dependent bending of a DNA duplex. These data revealed that the DNA distortions induced by enzyme binding and initial-complex formation depend on the nature of the damaged nucleotide. Molecular dynamics simulations showed that the damaged nucleotide is everted from the DNA helix and placed into the enzyme’s binding pocket. Nevertheless, no damage-specific contacts were detected between a damaged nucleotide and amino acid residues in the active site of the enzyme. It was suggested that the capacity of a damaged nucleotide to be everted from DNA and to be placed into the enzyme pocket could be the key factor behind the substrate specificity of APE1. This conclusion is in good agreement with the ability of APE1 to recognize many structurally unrelated nucleotides not only in DNA but also in RNA. According to the proposed model, one can conclude that the endoribonuclease activity of APE1 is linked with the structural features of RNA, which can facilitate the eversion of intact nucleotides in structurally distorted regions, such as a junction of stems and loops in hairpins, bulges, and bubbles. A plausible extrapolation of this model of substrate recognition suggests that APE1 can cleave not only RNA but also DNA in non-canonical B-forms. Therefore, the main objective of the present study was to obtain evidence for this supposition and to elucidate the key steps of the mechanism underlying APE1 interaction with model substrates that ensure specific recognition of a target nucleotide in DNA and RNA of non-canonical structures. To this end, we analyzed the efficacy of cleavage of a set of damaged DNA and native RNA substrates with non–B-form structures such as G-quadruplexes, hairpins, bulges, and bubbles that could facilitate the base eversion from the substrate into the APE1 active site.

## Materials and Methods

### The Enzyme and DNA and RNA Substrates

The APE1 enzyme was isolated from *Escherichia coli* Rosetta 2 cells transformed with plasmid pET11a carrying the human *APE1* gene (Daviet et al., 2007). Briefly, to purify APE1 expressed as a recombinant protein, 1 L of culture [in Luria–Bertani (LB) broth] of *E. coli* strain Rosetta II (DE3) (Invitrogen, France) carrying the pET11a-APE1 construct was grown with 50 μg/ml ampicillin at 37°C until absorbance at 600 nm (A600) reached 0.6–0.7; APE1 expression was induced overnight with 0.2 mM isopropyl-β-*d*-thiogalactopyranoside. The method for isolation of wild-type APE1 has been described previously (Kuznetsova et al., 2014; Miroshnikova et al., 2016a). The protein concentration was measured by the Bradford method; the stock solution was stored at −20°C.

The synthesis of the oligonucleotides (Table 1) was carried out on an ASM-800 DNA/RNA synthesizer (Biosset, Russia) using standard commercial phosphoramidites and CPG solid supports from Glen Research (United States). The oligonucleotides were deprotected according to manufacturer’s protocols and purified by HPLC. Oligonucleotide homogeneity was checked by denaturing 20% polyacrylamide gel electrophoresis (PAGE). Concentrations of oligonucleotides were calculated from their A_260_. Oligonucleotide duplexes were prepared by annealing oligonucleotide strands at a 1:1 molar ratio.

**TABLE 1:** Oligonucleotide sequences forming non-canonical DNA and RNA substrates.

**Folding type**	**Shorthand**	**Sequences**
G-quadruplex	Q4	5′-FAM-TTAGGGTTAGGGTTAGGGTTAGGGTT-BHQ1-3′
	F14-Q4	5′-FAM-TTAGGGTTAGGGTFAGGGTTAGGGTT-BHQ1-3′
	F14-aPu13-Q4	5′-TTAGGGTTAGGG(aPu)FAGGGTTAGGGTT-3′
	F14-aPu15-Q4	5′-TTAGGGTTAGGGTF(aPu)GGGTTAGGGTT-3′
	F17-Q4	5′-FAM-TTAGGGTTAGGGTTAGFGTTAGGGTT-BHQ1-3′
	F17-aPu16-Q4	5′-TTAGGGTTAGGGTTA(aPu)FGTTAGGGTT-3′
	F17-aPu18-Q4	5′-TTAGGGTTAGGGTTAGF(aPu)TTAGGGTT-3′
	rQ4	5′-FAM-r(AGGGUUAGGGUUAGGGUUAGGGU)-3′
Duplex	dsF/G	5′-FAM-GCGCATACGGCATFATCAGGGAAGTGGG-BHQ1-3′/3′-CGCGTATGCCGTAGTAGTCCCTTCACCC-5′
	rAUA/UAU	5′-FAM-r(GCGCAUACGGAAUAAAAGGGAAGUGGG)-3′/3′-r(CGCGUAUGCCUUAUUUUCCCUUCACCC)-5′
Bulged structures	F/−Δ1	5′-FAM-GCGCATACGGCATFATCAGGGAAGTGGG-BHQ1-3′/3′-CGCGTATGCCGTA-TAGTCCCTTCACCC-5′
	F/−Δ2(5′)	5′-FAM-GCGCATACGGCATFATCAGGGAAGTGGG-BHQ1-3′/3′-CGCGTATGCCGT–TAGTCCCTTCACCC-5′
	F/−Δ2(3′)	5′-FAM-GCGCATACGGCATFATCAGGGAAGTGGG-BHQ1-3′/3′-CGCGTATGCCGTA–AGTCCCTTCACCC-5′
	F/−Δ3	5′-FAM-GCGCATACGGCATFATCAGGGAAGTGGG-BHQ1-3′/3′-CGCGTATGCCGT—AGTCCCTTCACCC-5′
	F/−Δ5	5′-FAM-GCGCATACGGCATFATCAGGGAAGTGGG-BHQ1-3′/3′-CGCGTATGCCG—–GTCCCTTCACCC-5′
	F/ + Δ3	5′-FAM-GCGCATACGGCAT-F-ATCAGGGAAGTGGG-BHQ1-3′/3′-CGCGTATGCCGTAGGGTAGTCCCTTCACCC-5′
	F/ + Δ4	5′-FAM-GCGCATACGGCAT-F–ATCAGGGAAGTGGG-BHQ1-3′/3′-CGCGTATGCCGTACGGGTAGTCCCTTCACCC-5′
	F/ + Δ5	5′-FAM-GCGCATACGGCAT–F–ATCAGGGAAGTGGG-BHQ1-3′/3′-CGCGTATGCCGTACGGGCTAGTCCCTTCACCC-5′
	F/ + Δ7	5′-FAM-GCGCATACGGCAT—F—ATCAGGGAAGTGGG-BHQ1-3′/3′-CGCGTATGCCGTACCGGGCCTAGTCCCTTCACCC-5′
	rAUA/−Δ1	5′-FAM-r(GCGCAUACGGAAUAAAAGGGAAGUGGG)-3′/3′-r(CGCGUAUGCCUU-UUUUCCCUUCACCC)-5′
	rAUA/−Δ2	5′-FAM-r(GCGCAUACGGAAUAAAAGGGAAGUGGG)-3′/3′-r(CGCGUAUGCCUU–UUUCCCUUCACCC)-5′
	rAUA/−Δ3	5′-FAM-r(GCGCAUACGGAAUAAAAGGGAAGUGGG)-3′/3′-r(CGCGUAUGCCU—UUUCCCUUCACCC)-5′
Mismatch	rAUA/UCU	5′-FAM-r(GCGCAUACGGAAUAAAAGGGAAGUGGG)-3′/3′-r(CGCGUAUGCCUUCUUUUCCCUUCACCC)-5′
Bubbled structures	rAUA/CCC	5′-FAM-r(GCGCAUACGGAAUAAAAGGGAAGUGGG)-3′/3′-r(CGCGUAUGCCUCCCUUUCCCUUCACCC)-5′
Hairpin structure	rHP	5′-FAM-r(AUAUAACAUCAUUAUAU)-BHQ1-3′

### Circular Dichroism (CD) Analysis

These spectra were recorded on a Jasco J-600 spectropolarimeter (Jasco, Japan), at 25°C in quartz cells with 1 cm path length. The concentration of DNA in the cell was 10 μM. The experiments were carried out in a buffer consisting of 50 mM Tris-HCl pH 7.5, 140 mM KCl, 1 mM EDTA, and 5 mM MgCl_2_. The spectra were recorded at a bandwidth of 1.0 nm and a resolution of 1.0 nm at a scan speed of 50 nm/min.

### PAGE Experiments

5′-6-carboxyfluorescein (FAM)–labeled or ^32^P-labeled oligonucleotides were subjected to experiments on separation of cleavage products by PAGE. APE1 endonuclease assays with G-quadruplex DNA substrates were performed at 25°C in a reaction buffer consisting of 50 mM Tris-HCl pH 7.5, 5.0 mM MgCl_2_, and 140 mM KCl, which is required for quadruplex structure formation. In case of bulged DNA substrates, the concentration of KCl was reduced to 50 mM. In case of RNA substrates, experiments were conducted at 25°C in a reaction buffer consisting of 50 mM Tris-HCl pH 7.5, 50 mM KCl, and 1.0 mM EDTA to prevent 3′–5′ exonuclease degradation of substrates (Kuznetsova et al., 2020). The reaction was initiated by the addition of APE1. Aliquots of the reaction mixture were withdrawn, immediately quenched with a gel-loading dye containing 7 M urea and 25 mM EDTA, and loaded on a 20% (w/v) polyacrylamide/7 M urea gel. PAGE (gel concentration, 20%) was performed under denaturing conditions (7 Ì urea) at 55°C and a voltage of 200–300 V.

Partial hydrolysis of the model RNA substrates by RNase A was performed using the following procedure. The reaction mixture (20 μl) composed of a 3.0 μÌ substrate and 3.0 nM RNase A in a buffer [50 mM Tris-HCl (pH 8.5), 50 mM NaCl, 1 mM EDTA, 1 mM DTT, and 9% of glycerol] was incubated at 25°C for 5 min. The reaction mixture was then supplemented with 20 μl of a solution containing 7 M urea and 25 mM EDTA and incubated at 96°C for 5 min before gel loading.

Formation of the product was analyzed by autoradiography and quantified by scanning densitometry in the Gel-Pro Analyzer software, v.4.0 (Media Cybernetics, United States).

Kinetic traces of product accumulation, obtained in the PAGE analysis, were fitted to the single exponential curve by means of the Origin software (OriginLab Corp., United States; Eq. 1).

(1) Productaccumulation=A×[1-exp(-k×obst)]

where A is the amplitude, *k*_obs_ (s^–1^) denotes the observed rate constant, and *t* represents reaction time.

The dependence of observed rate constants *k*_obs_ on concentration of APE1 was fitted to Eq. 2:

(2) k=obsK×bindk×2[APE1]/(K×bind[APE1]+1)+k-2

where *K*_bind_ is the equilibrium constant of the initial APE1–substrate complex (M^–1^) and *k*_2_ and *k*_–2_ are the rate constants (s^–1^) of the second binding step.

In case of bulged DNA substrates, the initial rate of cleavage was estimated as the initial slope of the kinetic curve obtained under steady-state reaction conditions.

### Stopped-Flow Fluorescence Measurements

These measurements with fluorescence detection were carried out using an SX.18MV stopped-flow spectrometer (Applied Photophysics Ltd., United Kingdom) equipped with a 150 W Xe arc lamp and an optical cell with 2 mm path length. The dead time of the instrument is 1.4 ms. The fluorescence of Trp was excited at λ_ex_ = 290 nm and monitored at λ_em_ > 320 nm as transmitted by filter WG-320 (Schott, Mainz, Germany). If 2-aminopurine (aPu) was present in an oligonucleotide, λ_ex_ of 310 nm was chosen to excite aPu fluorescence, and its emission was monitored at λ_em_ > 370 nm (Corion filter LG-370). Fluorescence of a FAM residue was excited at λ_ex_ = 494 nm and monitored at λ_em_ > 515 nm as transmitted by filter OG-515 (Schott, Mainz, Germany). Stopped-flow fluorescence measurements were conducted with catalytically active APE1 at 25°C in a buffer consisting of 50 mM Tris-HCl pH 7.5, 140 mM KCl, and 5.0 mM MgCl_2_.

APE1 was placed in one of the instrument’s syringes and rapidly mixed in the reaction chamber with the DNA substrate from another syringe. The reported concentrations of reactants are those in the reaction chamber after the mixing. Typically, each trace shown in the figures is the average of four or more fluorescence traces recorded in individual experiments.

The sets of kinetic curves obtained at different concentrations of the reactants were analyzed in the DynaFit software (BioKin, Pullman, WA) (Kuzmic, 1996) as described elsewhere (Kladova et al., 2018b).

### Molecular Interaction Assays by Microscale Thermophoresis (MST)

Substrate-binding constants were determined via the MST approach on Monolith NT.115 (NanoTemper Technologies, Germany). The concentration of an oligonucleotide in all titration experiments was 0.5 μM, and concentrations of APE1 were varied from 0.05 to 30.0 μM. The reaction mixtures were incubated at 25°C in a buffer consisting of 50 mM Tris-HCl pH 7.5, 140 mM KCl, and 5 mM EDTA in case of G-quadruplexes and in a buffer consisting of 50 mM Tris-HCl pH 7.5, 50 mM KCl, and 5 mM EDTA in case of bulged duplexes. The values of the binding constant were calculated by means of Eq. 3:

(3) MSTsignal=F+noisF×amp[APE1]/(1/K+bind[APE1])

where F_nois_ is a background signal, F_amp_ is amplitude of an MST signal change, and *K*_bind_ is the equilibrium binding constant (M^–1^) for the formation of an enzyme–substrate complex.

In some cases, the signal-to-noise ratio was not sufficient for precise calculation of the binding constants. Therefore, all obtained values were used only as an evaluation of the ability of the enzyme to bind these structures.

## Results and Discussion

### Design of Model Substrates

**FIGURE 1:**
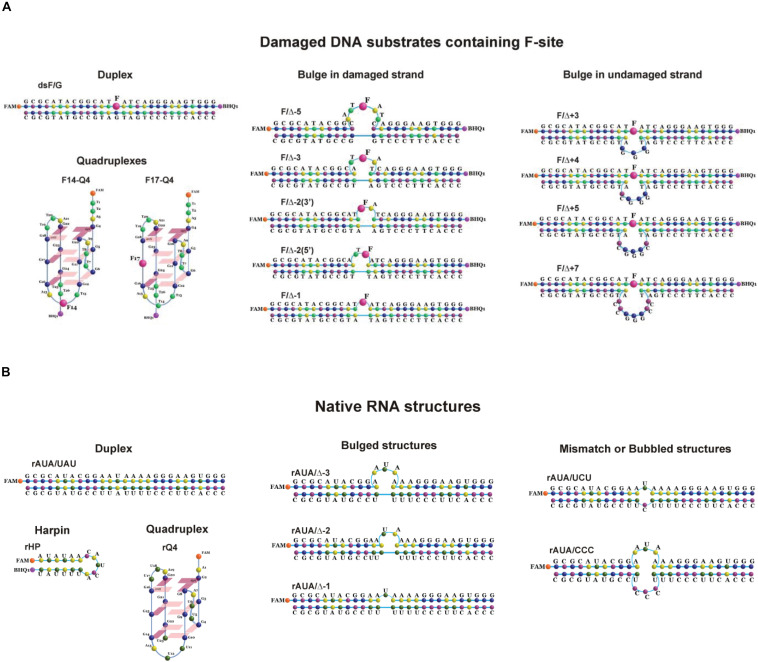
Structures of DNA **(A)** and RNA **(B)** substrates: schematic structures of a control DNA duplex (containing an F-site) **(A)** or a native RNA duplex **(B)**; the unimolecular human telomeric G-quadruplex (containing an F-site) **(A)** or a native RNA quadruplex **(B)**; DNA duplexes containing an F-site with bulging of a damaged (1–5 nucleotides) or undamaged (3–7 nucleotides) strand **(A)**; native RNA with bulged or bubbled structures (1–3 nucleotides) **(B)**; short hairpin RNA structure with a 6-nucleotide stem pair and 5 nucleotides in the loop region **(B)**.

According to our previous findings (Kuznetsova et al., 2018b), the substrate specificity of APE1 to damaged nucleotides in the duplexes is controlled by the ability of a given damaged nucleotide to be everted from the DNA double chain in response to an enzyme-induced DNA distortion. Therefore, in the present study, several non–B-form DNA and RNA structures were designed that can affect the nucleotide eversion process (Table 1). All the DNA and RNA structures used belong to one of several groups: (1) duplexes, (2) G-quadruplexes, (3) bulge-containing structures, (4) mismatch and bubble-containing structures, and (5) hairpins (Figure 1). In the case of DNA substrates, a stable analog of a natural AP site (F-site), lacking the hydroxyl group on the C1′-atom of ribose was utilized as a damaged nucleotide. RNA substrates contained only native undamaged nucleotides. A set of undamaged DNA oligonucleotides served as controls of the endonuclease and 3′–5′ exonuclease activities of APE1 (Table 1). A FAM residue was introduced into the 5′ end of oligonucleotides to visualize cleavage products on the polyacrylamide gel. Some oligonucleotides contained 3′-terminal black hole quencher (BHQ1) to create a FRET pair with the FAM residue for the assay of substrate cleavage by the stopped-flow kinetic method.

### Cleavage of Damaged DNA Substrates Having a Non–B-Form Structure

#### Cleavage of an F-Site in the DNA Forming G-Quadruplexes

The human telomeric G-quadruplex was employed as a model of a non-canonical DNA structure. Structural features of G-quadruplexes formed by undamaged oligonucleotides of the same sequence in both Na^+^ and K^+^ solutions have been reported elsewhere (Parkinson et al., 2002; Ambrus et al., 2006; Phan et al., 2007; Burra et al., 2019; Marzano et al., 2020). CD is usually used to study G-quadruplex DNA folding (Karsisiotis et al., 2011). There are three common topologies based on the directionality of neighboring strands, namely, parallel, antiparallel, and hybrid, each having a characteristic CD spectrum (Dai et al., 2007; Bugaut and Alberti, 2015).

Formation of the G-quadruplex structure by the Q4 sequence containing four TTAGGG telomeric repeats (Table 1) under the experimental conditions was confirmed by CD spectroscopy (Supplementary Figure S1A). The CD spectrum of unlabeled Q4 (Supplementary Figure S1B) reveals the hybrid type of its folding with a maximum at 295 nm. At the same time, the FAM-labeled Q4 quadruplex has greater amplitude at 265 nm in the CD spectra; this finding supports a parallel-type topology of the quadruplex. As expected, incorporation of an F-site into the loop region of the quadruplex (F14–Q4) does not cause significant changes in the CD spectrum. Furthermore, the CD spectrum of F17–Q4, which contains an F-site in the G-core region, also is very similar to the spectrum of Q4, thereby supporting the formation of the quadruplex structure by damaged oligonucleotides F14–Q4 and F17–Q4.

**FIGURE 2:**
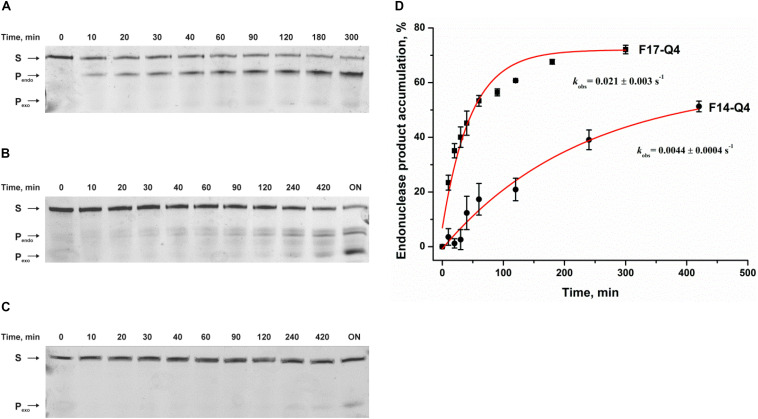
Endonuclease activity of APE1 toward a damaged DNA quadruplex substrate. PAGE analysis of cleavage of F14–Q4 **(A)**, F17–Q4 **(B)**, or undamaged Q4 **(C)** by APE1. **(D)** Time course of product accumulation. Concentrations of the DNA substrate and APE1 were 1.0 and 3.0 μM, respectively. “ON” means overnight incubation. S corresponds to 26-nt oligonucleotide forming a quadruplex structure; P_endo_ is a 13-nt or 16-nt product of cleavage of F14–Q4 and F17–Q4, respectively; and P_exo_ is the short products of exonuclease degradation.

PAGE analysis of product accumulation during interactions of APE1 with FAM/BHQ1-labeled F17–Q4 and F14–Q4 uncovered F-site cleavage in both cases (Figures 2A,B). Moreover, the interaction of APE1 with both damaged quadruplexes and undamaged Q4 leads to slow 3′–5′ exonuclease degradation of DNA (Figure 2C). These data indicated that APE1 can recognize and process an F-site both at the core and in the loop of a G-quadruplex structure with parallel-type topology, and does it with fivefold different efficiency as estimated by exponential fitting using Eq. 1 (*k*_obs_ = 0.0044 ± 0.0004 s^–1^ in the loop and 0.021 ± 0.003 s^–1^ at the core, respectively, Figure 2D). These data are consistent with and complement the detailed research (Zhou et al., 2015), suggesting that APE1 cleaves an F-site in a Na^+^-coordinated antiparallel topology (an F-site in the middle of the core GGG) but not in a K^+^-coordinated hybrid topology (the F-site on the 5′ side of the core GGG) of a telomeric quadruplex. Moreover, in a recent study (Burra et al., 2019), it was clearly demonstrated that placement of an F-site at different positions of the core region of a telomeric quadruplex leads to a significant change in the native hybrid conformation. It was found that a damaged quadruplex with a predominant parallel conformation is preferable for enzymatic digestion (Burra et al., 2019). Taken together, our results lead to the conclusion that the efficacy of the F-site cleavage is dependent on the folding topology of a quadruplex; this topology can be associated with both the location of the F-site within the core GGG sequence and Na^+^/K^+^ experimental conditions. Moreover, F-site cleavage in the loop region is less efficient than that of the core sequence.

To rule out that this low cleavage efficiency of damaged quadruplexes is associated with insufficient complex formation under the experimental conditions used, an MST assay was carried out to evaluate the binding constant between APE1 and the quadruplex substrates (Supplementary Figures S2, S3). Of note, the titration curve for the F14–Q4 substrate recorded by MST did not allow us to determine *K*_bind_ by this method because of a low signal-to-noise ratio. Nevertheless, MST assay revealed binding of Q4 and F17–Q4 substrates by APE1 with very close affinity (0.4 ± 0.2 and 0.5 ± 0.2 μM^–1^ for Q4 and F17–Q4, respectively).

**FIGURE 3:**
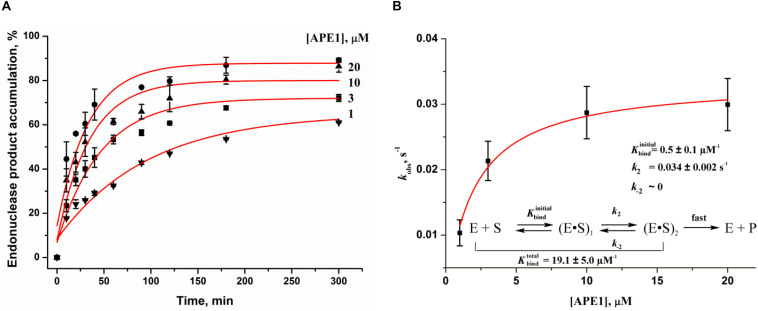
Effects of APE1 concentration on the F17–Q4 cleavage. **(A)** Accumulation of the reaction product as determined by PAGE. [F17–Q4] = 1.0 μM, final concentrations of APE1 are shown near the kinetic trace. **(B)** Dependences of the observed rate constants *k*_obs_ on APE1 concentration. The data were fitted to a hyperbolic equation Eq. 2.

To further evaluate DNA binding and catalytic steps of APE1 interaction with F17–Q4, the observed rate constants at different APE1 concentrations were determined (Figure 3A). The observed rate constant *k*_obs_ indicated a hyperbolic type of dependence on the APE1 concentration (Figure 3B), which corresponds to a two-step kinetic scheme with fast equilibrium initial substrate binding and can be fitted to Eq. 2. The equilibrium constant of the initial enzyme–substrate complex *K*_bind_ was estimated to be 0.5 ± 0.1 μM^–1^, indicating good binding of APE1 to the damaged quadruplex at the earliest stage of interaction. The rate constant of the second step, *k*_2_, was equal to 0.034 ± 0.002 s^–1^, whereas *k*_–__2_ was near zero, revealing that the slow second step should increase the total binding constant. It is known that the rate constant of F-site cleavage in duplex substrates is fast and varies from 0.3 to 5.5 s^–1^ (Kanazhevskaya et al., 2010, 2012; Timofeyeva et al., 2011; Miroshnikova et al., 2016a,b), 68 to 97 s^–1^ (Timofeyeva et al., 2009), or even 700–850 s^–1^ (Maher and Bloom, 2007; Schermerhorn and Delaney, 2013) depending on DNA duplex sequences, buffering conditions, and methods of cleavage detection. Therefore, the obtained rate constant of the second step is at least 10-fold less than the catalytic rate constant, suggesting that catalytic complex formation in the case of a quadruplex substrate is the rate-limiting step of the DNA cleavage (Figure 3B).

Because the recognition of a specific site in the substrate is accompanied by conformational adjustment of APE1 and DNA to optimize specific contacts in the enzyme–substrate complex, we performed a pre–steady-state kinetic analysis of conformational changes of APE1 and quadruplexes in the course of complex formation.

Real-time conformational rearrangements of APE1 and DNA during their interactions were visualized by stopped-flow kinetic assays with detection of intrinsic fluorescence from Trp residues of APE1 or fluorescence reporters incorporated into DNA, e.g., aPu and the FAM/BHQ1 FRET pair. The change in Trp fluorescence intensity in the reaction of APE1 with the DNA duplex containing an F-site consisted of characteristic phases (Supplementary Figure S4A) that have been identified earlier (Timofeyeva et al., 2009) as stages of DNA binding and catalysis. The decrease in fluorescence intensity in the initial part of the kinetic curves matches the formation of a catalytically competent complex. The catalytic stage of the process causes the formation of products and subsequent dissociation of the enzyme–product complex, which is accompanied by an increase in Trp fluorescence intensity at later time points (starting from approximately 0.1 s, Supplementary Figure S4A). As evidenced by the kinetic curves (Supplementary Figure S4A), the interaction of APE1 with a damaged or undamaged quadruplex does not result in any changes in the Trp fluorescence intensity, thereby strongly supporting the PAGE finding that the formation of the catalytic complex is slower than the cleavage reaction. In this case, detection of the catalytic complex by Trp fluorescence failed due to an insufficient concentration of this complex in the reaction mixture. On the other hand, these data can indicate that the type of binding of Q4 structures is different from the binding of DNA duplexes and therefore that the binding with quadruplex does not lead to Trp fluorescence quenching.

**FIGURE 4:**
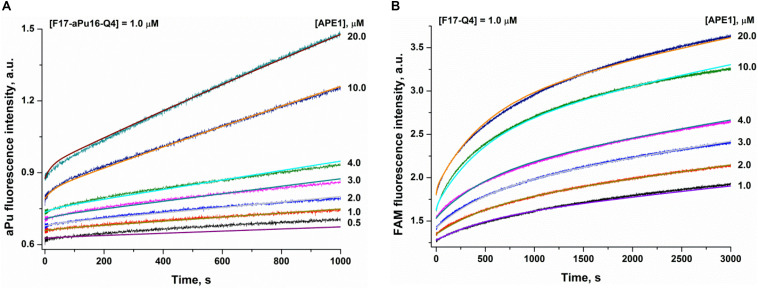
Interaction of APE1 with a damaged quadruplex containing an F-site at the 17th position. **(A)** aPu fluorescence kinetic traces (substrate F17–aPu16–Q4). **(B)** FAM/BHQ1 FRET kinetic traces (substrate F17–Q4). Jagged traces represent experimental data; smooth curves are the results of fitting to Scheme 1.

To analyze the substrate-binding process, a set of damaged quadruplexes was designed, containing an aPu residue (a fluorescent base analog) on the 3′ or 5′ side of an F-site in both substrates F14–Q4 and F17–Q4. Analysis of CD spectra revealed that the incorporation of aPu near the core F17 position (F17–aPu16–Q4 and F17–aPu18–Q4, Supplementary Figure S1B) results in preferable formation of a parallel type of quadruplex in comparison with the native hybrid fold of F14–aPu13–Q4 and F14–aPu15–Q4 structures, which contain modified nucleotides in the loop region. An analysis of fluorescence changes of aPu residues in all DNA substrates in the course of the interaction with APE1 (Supplementary Figure S4B) indicated that only F17–aPu16–Q4 was sensitive enough to detect enzyme–substrate complex formation. On the other hand, FRET detection allowed recording of the interaction of APE1 with either substrate F14–Q4 or F17–Q4 (Supplementary Figure S4C). A comparison of the profiles of fluorescence intensity among the interactions of APE1 with DNA quadruplexes containing an F-site allows us to select FAM/BHQ1-labeled F17–Q4 and F17–aPu16–Q4 as suitable models for further detailed analysis of the reaction with APE1 (Figure 4). Indeed, aPu fluorescence intensity is sensitive to the microenvironment of this residue and enabled registering of local conformational changes of a DNA substrate near the F-site (Figure 4A). On the other hand, the FAM/BHQ1-labeled F17–Q4 substrate helped to reveal “global” conformational changes that cause a change in the distance between the dye and quencher in the course of DNA substrate binding and cleavage (Figure 4B).

**SCHEME 1:**
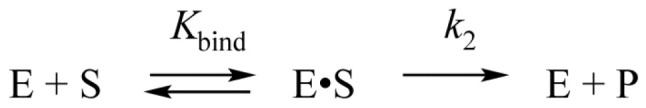
The kinetic mechanism of the interaction of APE1 with a damaged quadruplex. E, enzyme; S, substrate; ES, enzyme-substrate complex; P, product.

The rate constants of individual reaction steps (Table 2) were calculated via global fitting of the fluorescence data to Scheme 1, which contains the initial equilibrium binding step followed by the second rate-limiting transformation of the enzyme–substrate complex into the catalytic state in which a fast hydrolysis reaction takes place. The obtained data meant that different types of detection yield different values of initial substrate-binding constants *K*_bind_^initial^, indicating that initial transient complex ES detected by the different methods represents different “depths” of the damage recognition process. Nevertheless, all the methods of detection yielded equal rate constants *k*_2_ of the slow second binding step, which leads to catalytic complex formation. Altogether, these data indicated that the molecular processes that proceed at the second step of catalytic complex formation are crucial for damaged nucleotide recognition and therefore that facilitation of this step will significantly increase enzyme efficacy. Moreover, this step could be affected by the secondary structure of DNA.

**TABLE 2:** The rate and equilibrium constants of the interaction of APE1 with a damaged quadruplex.

**Constants**	**Type of detection**		
	**FRET**	**aPu**	**PAGE**
*K*_bind_^initial^, μM^–1^	0.08 ± 0.02	0.003 ± 0.001	0.5 ± 0.1
*k*_2_, s^–1^	0.03 ± 0.01	0.03 ± 0.01	0.034 ± 0.002

#### Cleavage of an F-Site in the Bulged DNA Structures

**FIGURE 5:**
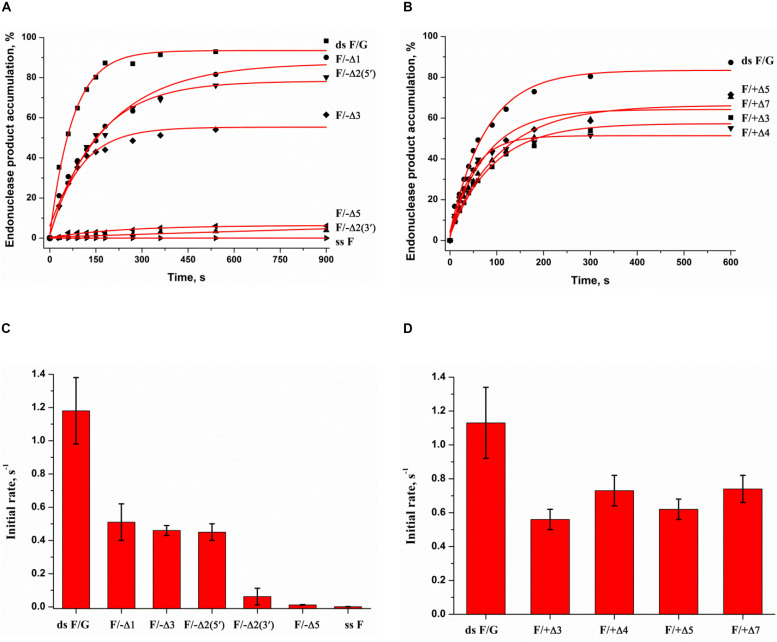
Endonuclease activity of APE1 toward bulged DNA substrates. Time course of product accumulation revealed by PAGE analysis of DNA substrates containing an F-site with bulging in the damaged **(A)** or undamaged **(B)** strand. Comparison of the cleavage efficacy of DNA substrates containing an F-site with bulging in the damaged **(C)** or undamaged **(D)** strand. Concentrations of the DNA substrate and APE1 were 1.0 and 20.0 nM, respectively.

To facilitate the rate-limiting second binding step of non–B-form substrates and to evaluate the effect of target nucleotide eversion in different structures, we designed a set of F-site – containing DNA duplexes with bulging of a damaged (1–5 nucleotides) or undamaged (3–7 nucleotides) strand (Table 1 and Figure 1B). The activity of APE1 was studied by direct PAGE analysis (Supplementary Figure S5), and the kinetics of accumulation of an endonucleolytic cleavage product were investigated (Figures 5A,B). The rate of DNA cleavage was estimated as the initial slope of the kinetic curve (Figures 5C,D).

It is noteworthy that the bulging of a single F-site (F/−Δ1) decreases the initial rate twofold in comparison with the full double-stranded substrate dsF/G (Figure 5C), suggesting that the substrate bending induced by the extra-helical position of the F-site slightly disturbs total interactions between the DNA-binding site of APE1 and the substrate. The constant of enzyme–substrate complex formation *K*_bind_ determined by MST (Supplementary Figure S2B) was also fourfold lower for F/−Δ1 in comparison with dsF/G. Moreover, an increase in bulging up to two F/−Δ2(5′) or three F/−Δ3 nucleotides does not have an additional effect on the enzymatic activity (Figure 5C) as well as substrate binding (Supplementary Figure S2B), keeping it at ∼50% of that of a fully complementary duplex. Therefore, expulsion of an abasic site from the double helix owing to bulge formation is not a key step in the process of F-site recognition.

Unexpectedly, the strongest effect on the enzymatic activity (Figure 5C), but not binding (Supplementary Figure S2B), was observed in the case of bulging to two nucleotides [F/−Δ2(3′), which contains an F-site near the 3′ end of the duplex stem in the substrate], thus supporting the conclusion that this location of the damaged nucleotide blocks catalytic-state formation. Analysis of the literature data revealed that bulged DNA and RNA duplexes can adopt a variety of conformations depending on the bulge size (Gohlke et al., 1994; Schreck et al., 2015; Strom et al., 2015). Consequently, the efficacy of cleavage of bulged damaged DNA depends both on the ability of a particular structure to eject the damaged nucleotide and also on the topological arrangement of the damaged nucleotide in this structure.

As depicted in Supplementary Figure S5G, APE1 does not hydrolyze an F-site in the single-stranded oligonucleotide ssF, indicating that APE1 cannot form appropriate contacts with the single-stranded substrate to adjust the damaged nucleotide in the active site. Accordingly, the loss of activity on the F/−Δ5 structure means that 5 bulged nucleotides completely mimic a single strand and do not allow the enzyme to form appropriate contacts with the substrate, whereas binding constant *K*_bind_ for F/−Δ5 has the highest value among the tested substrates (Supplementary Figure S2B). The bulging of 3–7 nucleotides in the undamaged strand opposite the F-site (Figure 5D) decreases enzymatic activity to ∼50% in comparison with a fully complementary duplex regardless of loop size. This effect most likely is associated with an interaction of the enzyme predominantly with the damaged strand of the substrate.

Thus, it could be assumed that bulging of 5 nucleotides in the damaged strand is the critical size of a single-stranded region for the recognition of an F-site in the DNA and for the formation of a catalytic complex. By contrast, the loss of the activity in the case of F/−Δ2(3′) suggests that the position of the F-site in the 2-nucleotide bulging sequence TF [F/−Δ2(5′)] or FA [F/−Δ2(3′)] is also crucial for catalytic complex formation. Independence of DNA cleavage from the loop size in the undamaged strand confirms the ability of the enzyme to place large nucleotide moieties outside the active site. These findings also support the notion that F-site cleavage in the quadruplexes proceeds without significant distortion of the quadruplex structure, with no more than 5 single-stranded nucleotides near the F-site and placement of a large undamaged part of the quadruplex structure outside of the active site, as demonstrated for a 3- to 7-nucleotide bulge of the complementary strand in the duplexes.

### Cleavage of Native RNA Substrates Having a Non–B-Form Structure

It has been reported earlier that APE1 has endoribonuclease activity (Barzilay and Hickson, 1995). As for the cleavage of undamaged RNA, APE1 preferentially catalyzes hydrolysis of the phosphodiester bond inside dinucleotides UA, UG, and CA in bulged sequences or weakly paired RNA regions (Berquist et al., 2008; Barnes et al., 2009; Kim et al., 2010), in good agreement with observed selectivity of APE1 toward damaged DNA substrates. A comparative analysis (Kuznetsova et al., 2020) of the cleavage efficiency of model RNA substrates containing short-hairpin structures, in which the loop size was varied from 2 to 5 nucleotides, has revealed that the 5-nucleotide-long hairpin loop is more readily adapted to the substrate-binding site of the enzyme than shortened versions. Moreover, the position of the cleavage site in the 5-nucleotide loop also influenced the hydrolysis efficacy. Recent findings (Antoniali et al., 2017) revealed that endoribonuclease activity of APE1 could play an important role in RNA metabolism. It was shown (Antoniali et al., 2017) that APE1 is required for the processing of microRNAs miR-221/miR-222 (Garofalo et al., 2011) and miR-92b (Sun et al., 2019), which are considered to act as tumor suppressors.

**FIGURE 6:**
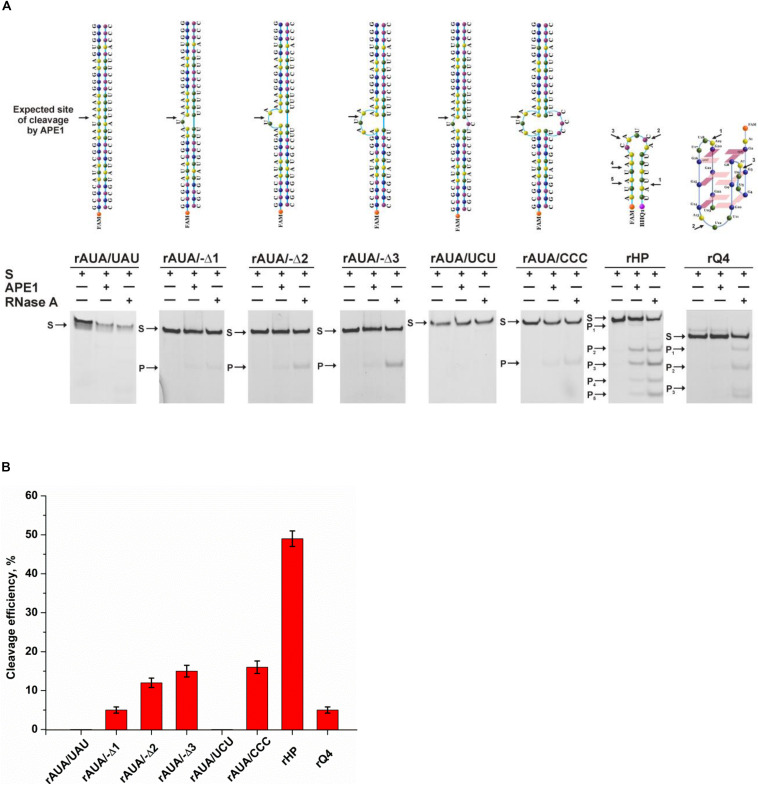
The efficiency of cleavage of RNA substrates by APE1. **(A)** PAGE analysis of the reaction products. Positions of the hydrolyzed nucleotides are pointed out by arrows. **(B)** Comparison of the efficacy of cleavage of RNA substrates by APE1. [APE1] = 2 μM, [RNA] = 1 μM, reaction time = 1 h.

To verify the mechanism of target nucleotide selection proposed in the present study, a series of native RNA substrates with a non–B-form structure was tested (Figure 6). To identify the cleavage site of APE1, all the tested RNA substrates were treated with RNase A, which selectively cleaves pyrimidine nucleotides. As illustrated in Figure 6A, a fully complementary stable RNA duplex, rAUA/UAU, was not cleaved by APE1, and even the formation of a single mismatch, rAUA/UCU, does not permit the formation of a catalytic complex with APE1. Nevertheless, bulging of a single uridine leads to slight product formation. Of note, an increase of the bulge from 1 to 3 nucleotides was accompanied by a stepwise increase of cleavage activity up to 15% (Figure 6B). Moreover, the efficacy of cleavage of a bubbled structure containing three mispaired nucleotides was also near 15%, again revealing the independence of the activity from the size of a non-target strand of the substrate. A short-hairpin structure was cleaved with the highest efficacy among the tested structures, indicating that a 5-nucleotide loop is sufficient for effective catalytic complex formation. Furthermore, it can be assumed that after cleavage in the loop region, the stability of a short RNA duplex is not sufficient to protect it from subsequent hydrolysis of all pyrimidine–purine sites in this sequence. Summarized hydrolysis of all five possible target sites leads to 50% cleavage of substrate rHP. It is worth noting that 3-nucleotide loops in the quadruplex structure rQ4, which contains a UA context, were very resistant to APE1 action with summary cleavage efficiency up to 5%. Taken together, it could be concluded that APE1 more efficiently cleaves RNA which contains single-stranded regions as a bulge or loop located near the duplex part, supporting the findings that the biological significance of the endoribonuclease activity is associated with processing of RNA in such non-canonical structures.

**FIGURE 7:**
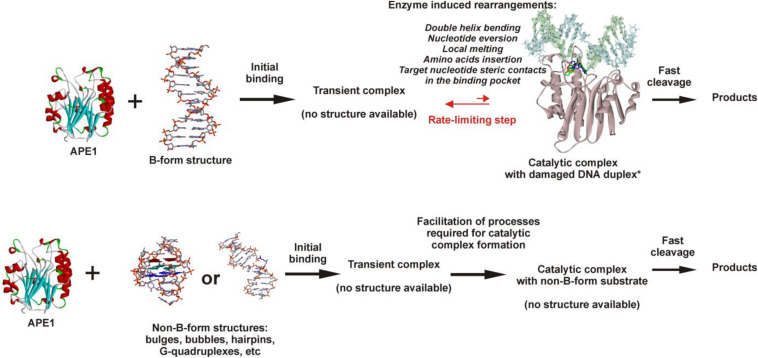
The proposed model of target nucleotide recognition by APE1. Structure of APE1–DNA complex from Kuznetsova et al. (2018b).

Considering our findings about the RNA substrates, it can be concluded that APE1 uses the same molecular mechanism of recognition of a target nucleotide in RNA substrates as the mechanism of F-site recognition in damaged DNA substrates. Moreover, the wide substrate specificity of APE1 toward an abasic site, some damaged bases [for example, etheno derivatives of DNA bases (Christov et al., 2010; Prorok et al., 2012), bulky photoproducts (Vrouwe et al., 2011), benzene-derived DNA adducts (Guliaev et al., 2004), α-anomers of 2′-deoxynucleosides (Gros et al., 2004), oxidatively damaged pyrimidines (Daviet et al., 2007), or 2′-deoxyuridine (Prorok et al., 2013)], and native deoxyribonucleotides (3′–5′ exonuclease activity) and ribonucleotides (endoribonuclease activity) strongly supports the conclusion that the active site of APE1 does not form direct specific contacts with a damaged or native target nucleotide. Consequently, the common mechanism of target nucleotide recognition by APE1 (Figure 7) includes the formation of an initial enzyme–substrate complex in which sequential conformational changes of the substrate in response to enzyme-induced interactions is strongly needed for the formation of a catalytically active complex. The molecular processes that take place in the course of these rearrangements, such as substrate bending, local melting, target nucleotide eversion from the substrate and insertion into the enzyme active site, amino acid insertion, and the formation of a network of contacts, are important for the target nucleotide recognition and limit the rate of catalytic complex formation. Moreover, it is known that the unstructured N-terminal domain of APE1 is the key part of the enzyme, which is responsible for the redox function (Evans et al., 2000; Kelley et al., 2011; Bazlekowa-Karaban et al., 2019), plays an important role in the interaction with damaged DNA and RNA (Timofeyeva et al., 2009; Fantini et al., 2010), and also affects the protein–protein interactions of APE1 with nucleophosmin (Poletto et al., 2013) and other participants of the BER pathway (Kladova et al., 2018a; Moor et al., 2020). Therefore, it could be assumed that the N-terminal domain of APE1 also can affect the ability of the enzyme to bind non–B-form structures and participate in the recognition of the target nucleotide.

It is reasonable to suggest that facilitation of some of the molecular events during binding processes, for example, the eversion of the target nucleotide or the bending of the substrate, because of the features of the initial structure of the substrate with non–B-form components, may increase the rate of substrate cleavage. It should be mentioned that in addition to the eversion of a target nucleotide from the substrate structure, its insertion into the active site should be taken into account too. Of note, literature data available today about the APE1 activity on B-form damaged DNA duplexes enable a comparison of the efficacy of cleavage among different damaged DNA duplexes and thereby a comparison of the rates of the limiting step. The recognition and cleavage of an F-site in the complementary duplex proceed during a 1 s period (Supplementary Figure S4A), whereas the duration of recognition of damaged nucleotides in duplexes—e.g., 1,*N*^6^-ethenoadenosine, α-adenosine, or 5,6-dihydrouridine (Kuznetsova et al., 2018b) – is longer, up to 1,000 s. Moreover, native ribonucleotides are processed by the enzyme within hours (Figure 6). It can be concluded that the nature of a target nucleotide that is inserted into the active site is also a very important factor for the formation of a catalytically active state of the APE1–substrate complex.

The obtained results mean that facilitation of F-site eversion in bulged structures decreases cleavage efficacy in comparison with a fully complementary duplex because of a decrease in the substrate-binding constant. During the eversion of a small abasic site and its insertion into the active site of the enzyme, the deoxyribose residue does not engage in any unfavorable interactions. By contrast, the insertion of any target nucleotide containing a damaged or native base into the active site of APE1 is much slower than the insertion of an abasic nucleotide because of steric hindrance. Indeed, in the case of bulged RNA, the cleavage efficacy clearly increased with the increasing bulge size, indicating that promotion of the eversion by the structure leads to skipping of some unfavorable contacts in the course of target base flipping out and insertion into the active site.

## Conclusion

Thus, a series of damaged DNA substrates and undamaged RNA substrates of a non–B-form structure was tested to evaluate the structural aberrations facilitating the process of target nucleotide recognition by APE1 and the formation of a catalytically competent complex. It was experimentally demonstrated that a human telomeric G-quadruplex containing an F-site, a native RNA quadruplex, and DNA duplexes containing an F-site with bulging of a damaged or undamaged strand as well as a native RNA having a bulged or bubbled structure and a short-hairpin RNA structure are processed during catalytic endonucleolytic cleavage by human AP endonuclease. It is important to note that in some specially designed DNA structures, a site-specific cleavage of a single-stranded DNA occurs as well. The mechanism of target nucleotide recognition is composed of two steps: initial transient complex formation and its subsequent rate-limiting transformation into a catalytically competent state. Facilitation of the eversion of a target nucleotide into the enzyme active site is mediated by some non–B-form regions in the substrate, which allows us to increase the formation rate of a catalytic complex, thereby enhancing the efficacy of the enzymatic catalysis. Thus, it is shown that the formation of non-canonical B-form structures by undamaged DNA is the key factor for the processing of a target nucleotide in the active site of the enzyme. In general, data obtained in the research on the specificity of AP endonuclease toward DNA and RNA substrates of various spatial structures expand our understanding of the molecular principles governing target nucleotide recognition by APE1.

## Data Availability Statement

All datasets presented in this study are included in the article/Supplementary Material.

## Author Contributions

AD and AK conducted the experiments. NK conceived and designed the experiments. AD, AK, NK, and OF analyzed the data. NK and OF contributed the reagents, materials, analytical tools, and wrote the manuscript. All authors contributed to the article and approved the submitted version.

## Conflict of Interest

The authors declare that the research was conducted in the absence of any commercial or financial relationships that could be construed as a potential conflict of interest.
