# Supplementary material for: Activity of Human Apurinic/Apyrimidinic Endonuclease APE1 Toward Damaged DNA and Native RNA With Non-canonical Structures
Source: Front Cell Dev Biol. 2020 Oct 30;8:590848. doi: 10.3389/fcell.2020.590848 (PMC7662432; doi:10.3389/fcell.2020.590848)
Supplement: Supplementary file 1 [file Data_Sheet_1.pdf]

Activity of human apurinic/apyrimidinic endonuclease APE1 towards damaged DNA and native RNA with non-canonical structure

Anastasia T. Davletgildeeva[1,2,†], Alexandra A. Kuznetsova[1,†], Olga S. Fedorova[1,*], Nikita A. Kuznetsov[1,*]

[1]Institute of Chemical Biology and Fundamental Medicine, Siberian Branch of Russian Academy of Sciences, Novosibirsk 630090, Russia

[2]Department of Natural Sciences, Novosibirsk State University, Novosibirsk 630090, Russia

* To whom correspondence should be addressed: O.S.F. Tel. +7(383)363-5174, Fax +7(383)363-5153, E-mail: fedorova@niboch.nsc.ru; N.A.K. Tel. +7(383)363-5174, Fax +7(383)363-5153, E-mail: nikita.kuznetsov@niboch.nsc.ru.

A B

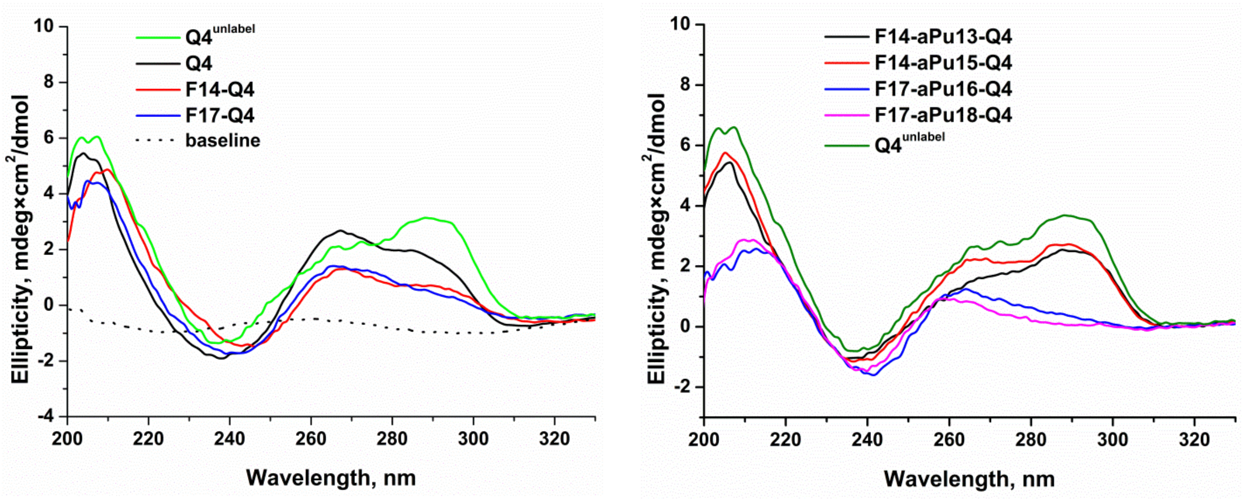

Fig. S1. CD spectra recorded for DNA oligonucleotides with a telomeric-repeat context. (A) Comparison of FAM-labelled quadruplexes Q4, F14-Q4 and F17-Q4 with a control unlabelled sequence, Q4. (B) Comparison of unlabelled Q4 and aPu-labelled quadruplexes F14-Q4 and F17-Q4. The CD spectra were acquired in the 50 mM Tris-HCl (pH 7.5) buffer containing 140 mM KCl and 5 mM $MgCl_2$ at 25°C.

A B

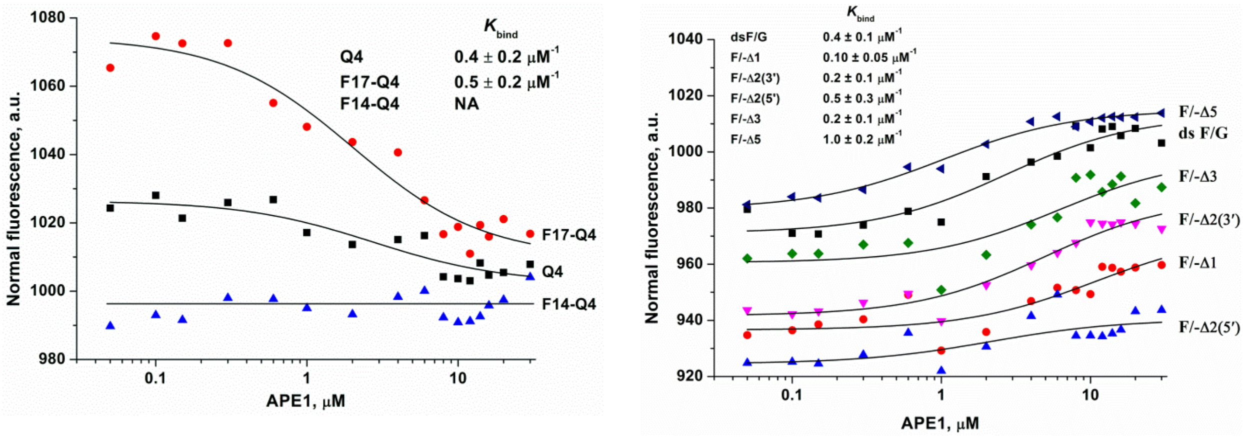

Fig. S2. Determination of the binding constant of enzyme–substrate complexes involving quadruplexes (A) and bulged duplexes (B) by MST.

A

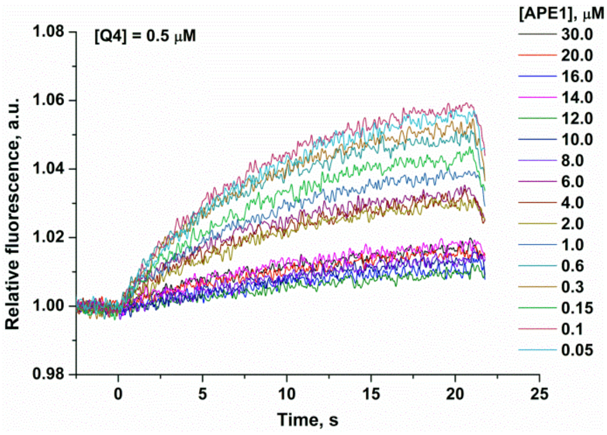
[Q4] = 0.5 μM
[APE1], μM
30.0
20.0
16.0
14.0
12.0
10.0
8.0
6.0
4.0
2.0
1.0
0.6
0.3
0.15
0.1
0.05
Relative fluorescence, a.u.
1.08
1.06
1.04
1.02
1.00
0.98
0
5
10
15
20
25
Time, s

B

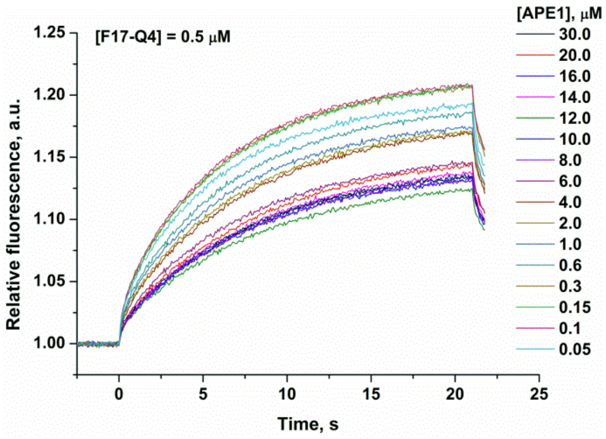
[F17-Q4] = 0.5 μM
[APE1], μM
30.0
20.0
16.0
14.0
12.0
10.0
8.0
6.0
4.0
2.0
1.0
0.6
0.3
0.15
0.1
0.05
Relative fluorescence, a.u.
1.25
1.20
1.15
1.10
1.05
1.00
0
5
10
15
20
25
Time, s

C

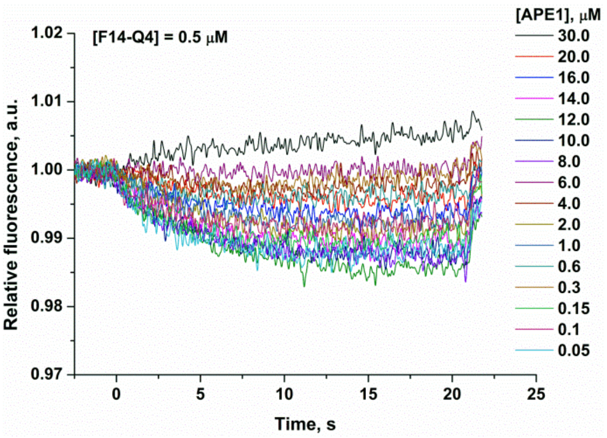
[F14-Q4] = 0.5 μM
[APE1], μM
30.0
20.0
16.0
14.0
12.0
10.0
8.0
6.0
4.0
2.0
1.0
0.6
0.3
0.15
0.1
0.05
Relative fluorescence, a.u.
1.02
1.01
1.00
0.99
0.98
0.97
0
5
10
15
20
25
Time, s

D

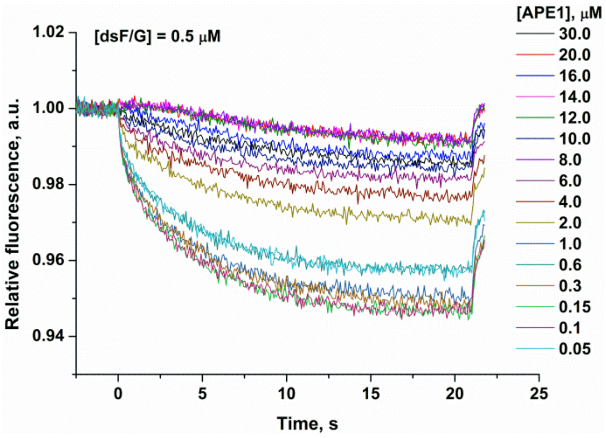
[dsF/G] = 0.5 μM
[APE1], μM
30.0
20.0
16.0
14.0
12.0
10.0
8.0
6.0
4.0
2.0
1.0
0.6
0.3
0.15
0.1
0.05
Relative fluorescence, a.u.
1.02
1.00
0.98
0.96
0.94
0
5
10
15
20
25
Time, s

E

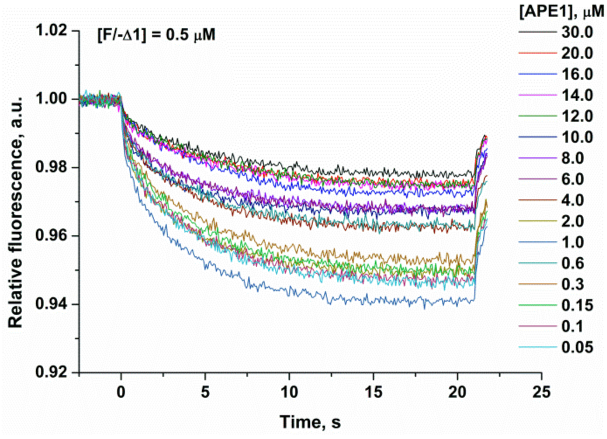
[F/-Δ1] = 0.5 μM
[APE1], μM
30.0
20.0
16.0
14.0
12.0
10.0
8.0
6.0
4.0
2.0
1.0
0.6
0.3
0.15
0.1
0.05
Relative fluorescence, a.u.
1.02
1.00
0.98
0.96
0.94
0.92
0
5
10
15
20
25
Time, s

F

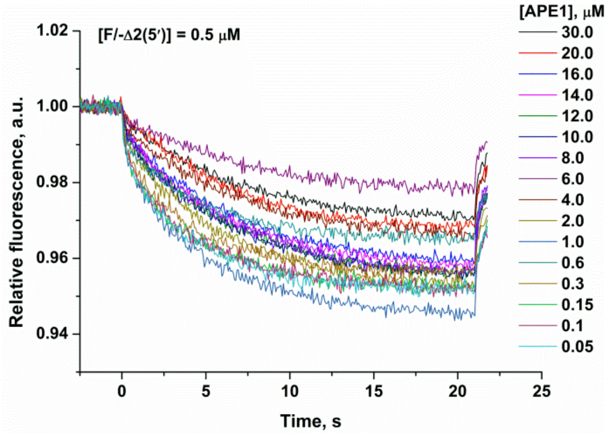
[F/-Δ2(5′)] = 0.5 μM
[APE1], μM
30.0
20.0
16.0
14.0
12.0
10.0
8.0
6.0
4.0
2.0
1.0
0.6
0.3
0.15
0.1
0.05
Relative fluorescence, a.u.
1.02
1.00
0.98
0.96
0.94
0
5
10
15
20
25
Time, s

J

H

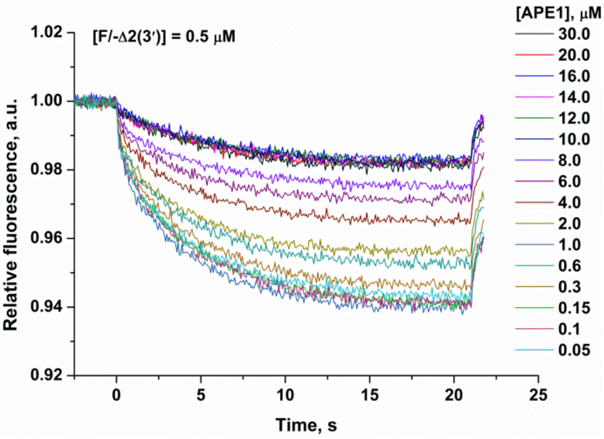

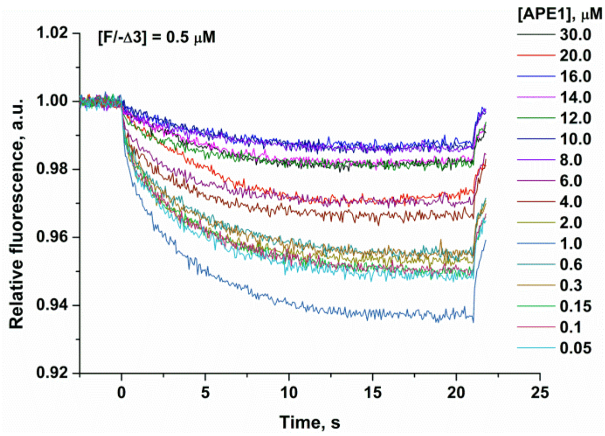

I

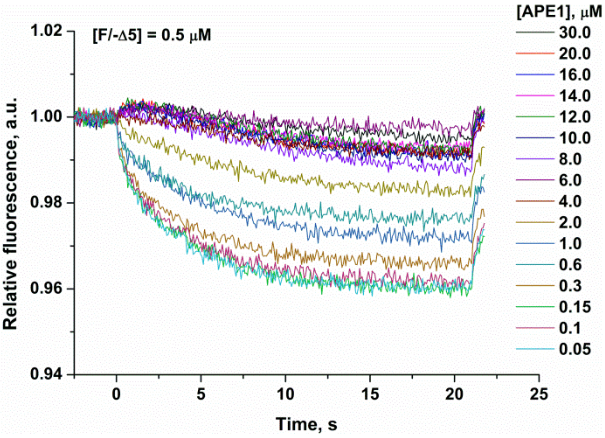

Fig. S3. MST thermograms for the binding interaction between DNA and APE1. (A) Q4, (B) F17-Q4, (C) F14-Q4, (D) dsF/G, (E) F/-Δ1, (F) F/-Δ2(5′), (J) F/-Δ2(3′), (H) F/-Δ3, (I) F/-Δ5.

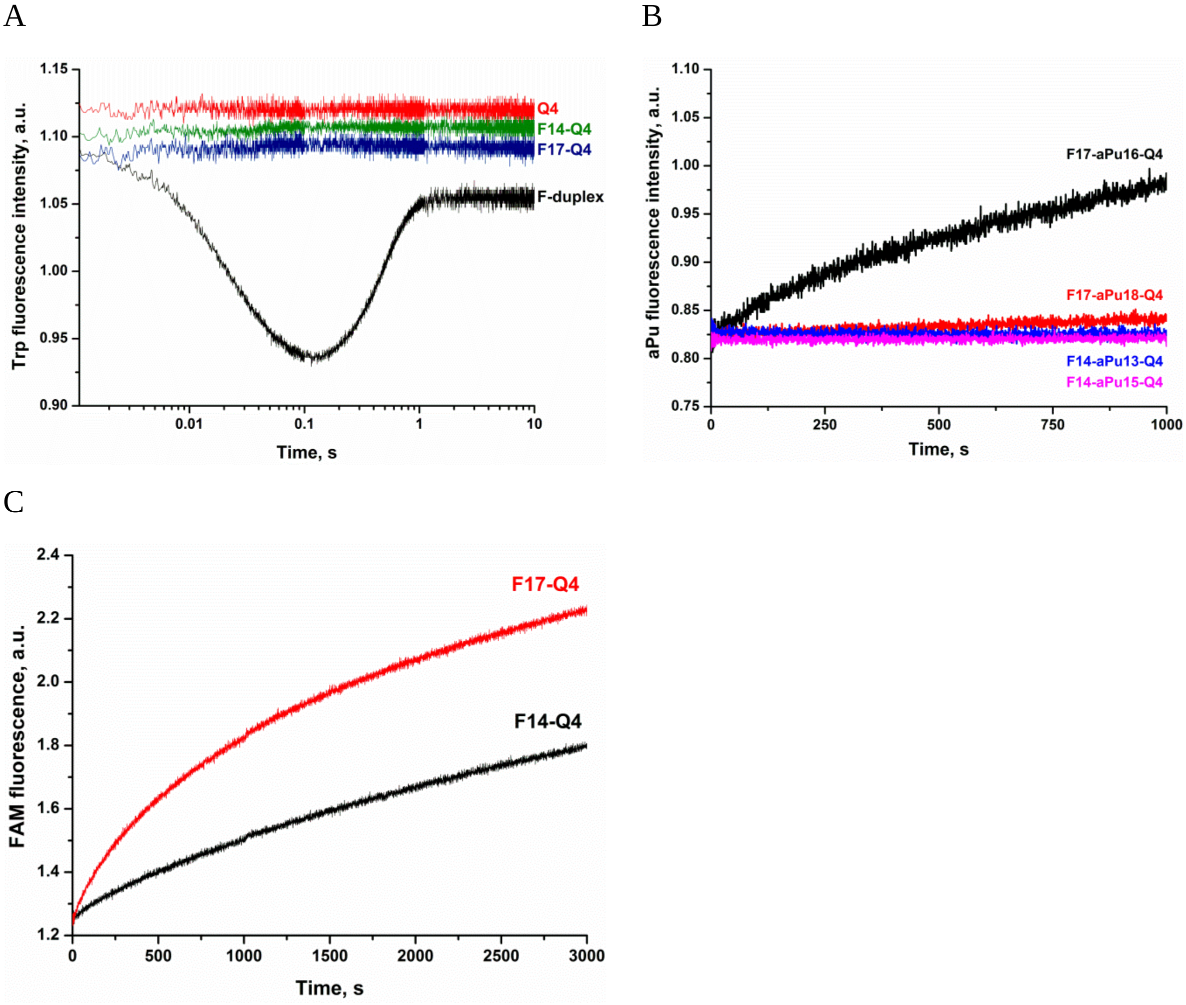

Fig. S4. A comparison of profiles of fluorescence intensity among the interactions of APE1 with DNA quadruplexes containing an F-site, as registered by means of fluorescence intensity of Trp (A), aPu (B) or FAM (C). [DNA] = 1.0 μM, [APE1] = 2.0 μM.

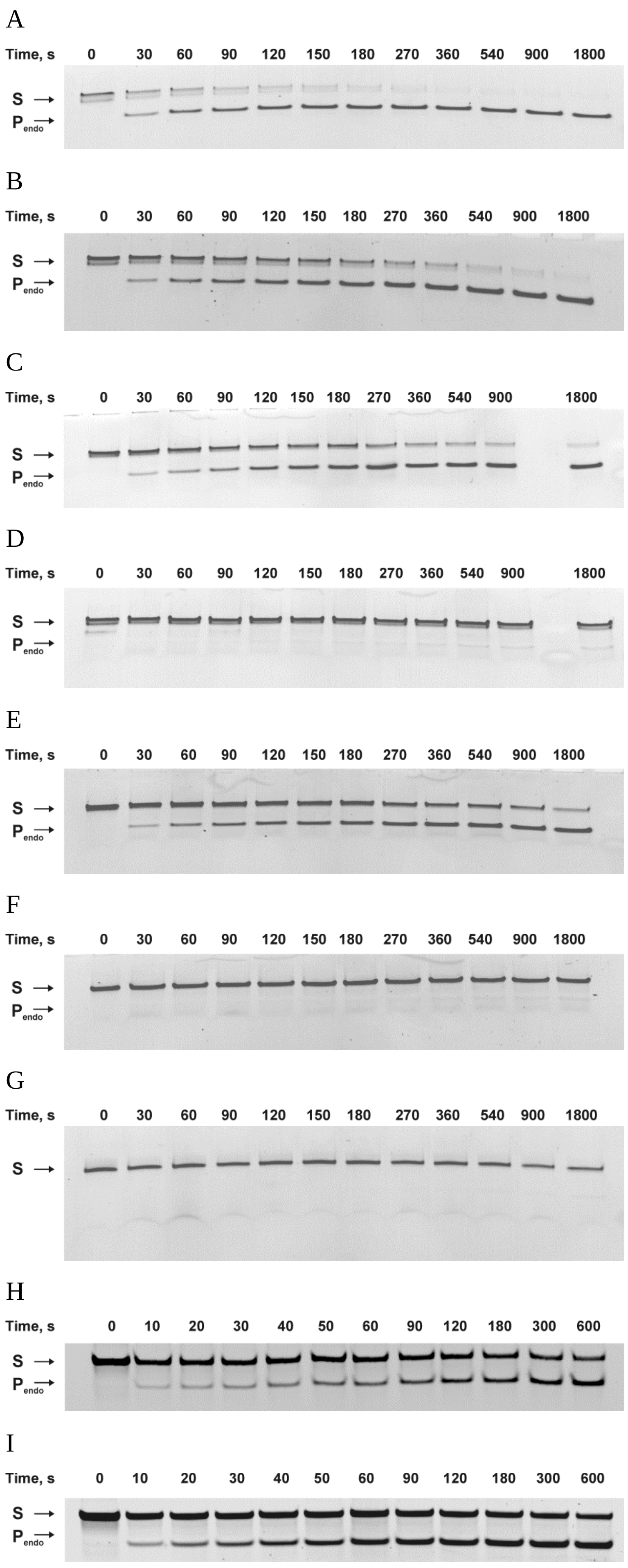
A
Time, s 0 30 60 90 120 150 180 270 360 540 900 1800
S
Pendo
B
Time, s 0 30 60 90 120 150 180 270 360 540 900 1800
S
Pendo
C
Time, s 0 30 60 90 120 150 180 270 360 540 900 1800
S
Pendo
D
Time, s 0 30 60 90 120 150 180 270 360 540 900 1800
S
Pendo
E
Time, s 0 30 60 90 120 150 180 270 360 540 900 1800
S
Pendo
F
Time, s 0 30 60 90 120 150 180 270 360 540 900 1800
S
Pendo
G
Time, s 0 30 60 90 120 150 180 270 360 540 900 1800
S
H
Time, s 0 10 20 30 40 50 60 90 120 180 300 600
S
Pendo
I
Time, s 0 10 20 30 40 50 60 90 120 180 300 600
S
Pendo

J

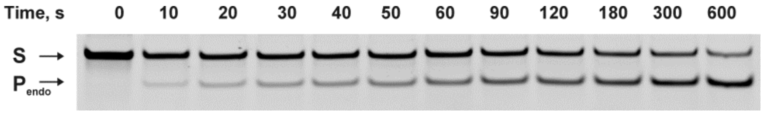

K

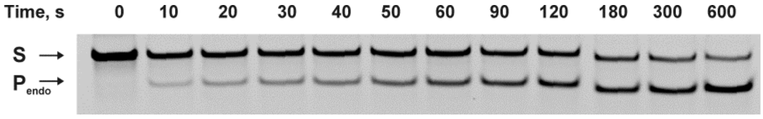

Fig. S5. Endonuclease activity of APE1 towards fully complement duplex dsF/G (A) and bulged structures F/-Δ1 (B), F/-Δ2(5′) (C), F/-Δ2(3′) (D), F/-Δ3 (E), F/-Δ5 (F), single stranded ssF (G), F/+Δ3 (H), F/+Δ4 (I), F/+Δ5 (J) and F/+Δ7 (K). [DNA] = 2.0 μM, [APE1] = 20 nM.

S corresponds to 28 nt oligonucleotide, P_{endo} is 13 nt product of cleavage of F-site containing DNA strand.
